# Delayed Cutaneous Microvascular Responses With Non-consecutive 3 Days of Remote Ischemic Preconditioning

**DOI:** 10.3389/fphys.2022.852966

**Published:** 2022-03-14

**Authors:** Jahyun Kim, Warren D. Franke, James A. Lang

**Affiliations:** ^1^Department of Kinesiology, California State University, Bakersfield, Bakersfield, CA, United States; ^2^Department of Kinesiology, Iowa State University, Ames, IA, United States

**Keywords:** laser speckle contrast imaging, skin blood flow, iontophoresis, local heating, endothelial dependent cutaneous vasodilation, endothelial independent cutaneous vasodilation, vascular adaptation, non-consecutive repeated remote ischemic preconditioning

## Abstract

The optimal frequency and duration of remote ischemic preconditioning (RIPC) that augments microvascular function is unknown. A single bout of RIPC increases cutaneous endothelial function for ∼48 h, whereas 1 week of daily RIPC bouts improves more sustained endothelium-independent function. We hypothesized that 3 days of RIPC separated by rest days (3QOD RIPC) would result in sustained increases in both endothelium-dependent and endothelium-independent functions. Cutaneous microvascular function was assessed in 13 healthy young participants (aged 20.5 ± 3.9 years; 5 males, 8 females) before 3QOD and then 24, 48, and 72 h and a week after 3QOD. RIPC consisted of four repetitions of 5 min of blood flow occlusion separated by 5 min of reperfusion. Skin blood flow responses to local heating (*T*_loc_ = 42°C), acetylcholine (Ach), and sodium nitroprusside (SNP) were measured using laser speckle contrast imaging and expressed as cutaneous vascular conductance (CVC = PU⋅mmHg^–1^). Local heating-mediated vasodilation was increased 72 h after 3QOD and the increased responsivity persisted a week later (1.08 ± 0.24 vs. 1.34 ± 0.46, 1.21 ± 0.36 PU⋅mmHg^–1^; ΔCVC, pre-RIPC vs. 72 h, a week after 3QOD; *P* = 0.054). Ach-induced cutaneous vasodilation increased a week after 3QOD (0.73 ± 0.41 vs. 0.95 ± 0.49 PU⋅mmHg^–1^; ΔCVC, pre-RIPC vs. a week after 3QOD; *P* < 0.05). SNP-induced cutaneous vasodilation increased 24 h after 3QOD (0.47 ± 0.28 vs. 0.63 ± 0.35 PU⋅mmHg^–1^; ΔCVC, pre-RIPC vs. 24 h; *P* < 0.05), but this change did not persist thereafter. Thus, 3QOD induced sustained improvement in endothelium-dependent vasodilation but was not sufficient to sustain increases in endothelium-independent vasodilation.

## Introduction

Ischemic heart disease and stroke are leading causes of death and have a high recurrence rate (Briffa et al., 2011; Benjamin et al., 2019). Regular moderate to high-intensity exercise training improves cardiovascular health and reduces cardiovascular disease incidence (Riebe et al., 2015). However, this intensity and frequency of exercise may not be practical in some clinical populations, such as those with severe ischemic heart disease or with limited mobility. Remote ischemic preconditioning (RIPC), consisting of three or four cycles of limb blood flow occlusion followed by reperfusion, may be an alternative intervention to improve cardiovascular function in these clinical populations. However, the threshold needed to elicit beneficial effects remains unclear. In the vasculature, a single bout of RIPC transiently increases endothelial function that peaks ∼48 h after RIPC (Loukogeorgakis et al., 2005; Kim et al., 2021); however, this stimulus may not be sufficient to substantively improve clinical outcomes (Hausenloy et al., 2015; Meybohm et al., 2015).

Previous studies indicate that repeated bouts of RIPC confer additional and long-lasting vascular benefits (Thijssen et al., 2016; Lang et al., 2019). Repeated RIPC augments the brachial flow-mediated dilation response, an index of conduit artery endothelial function (Moro et al., 2011; van den Munckhof et al., 2013; Jones et al., 2014). In the microvasculature, we found that repeated RIPC (i.e., 12 bouts) improves cutaneous endothelium-dependent and endothelium-independent vasodilation, which is an indicator of vascular smooth muscle function (Kim et al., 2020). However, these 12 bouts of RIPC did not further augment cutaneous endothelium-dependent vasodilation beyond that seen with 7 bouts of RIPC (Kim et al., 2020), and the improvement in cutaneous microvascular function observed only after 7 bouts of RIPC persisted for a week (Lang et al., 2019). In contrast, a single bout of RIPC increased endothelium-dependent vasodilation during 24–48 h after a bout of RIPC, but this effect was not sustained for 72 h after a bout of RIPC, and endothelium-independent vasodilation was not affected (Kim et al., 2021). Collectively, these studies indicate that although a single bout of RIPC is sufficient to increase endothelium-dependent vasodilation, repeated bouts of RIPC are required to induce sustained improvement in endothelium-dependent and endothelium-independent vasodilation.

Using the skin circulation as a microvascular model (Holowatz et al., 2008; Khan et al., 2008; Minson, 2010), we tested whether 3 days of RIPC separated by rest days (3QOD RIPC) improves microvascular function and induces persistent functional augmentation. Administering RIPC during the peak of the window of improvement (i.e., 48 h after or every other day) elicited by the previous RIPC bout may further enhance the beneficial effects of RIPC. Thus, multiple RIPC on this delayed window phase may induce strong sustainable microvascular function augmentation with less repeated RIPC bouts. Therefore, we hypothesized that 3QOD RIPC will induce sustained augmentation in endothelium-dependent vasodilation, reflected by augmented cutaneous responses to local heating and Ach-mediated, and in endothelium-independent vasodilation to sodium nitroprusside (SNP) for a week after the last bout of RIPC.

## Materials and Methods

### Subjects and Ethical Approval

Verbal and written informed consents were obtained from all participants before the experiments. The experiment procedure conformed to the standards set by the *Declaration of Helsinki* and was approved by the Institutional Review Board at Iowa State University (IRB# 19-482-00) and the Food and Drug Administration (FDA IND# 138343). A total of 13 healthy normotensive young subjects [aged 20.5 ± 3.9 years; 5 males, 8 females; mean arterial pressure (MAP) = 83.8 ± 6.0 mmHg; body mass index (BMI) = 22.9 ± 2.8 kg/m^2^] were involved in this study. Previous studies have shown that 13 participants were sufficient to see differences in RIPC-induced skin blood flow changes (Kim et al., 2020, 2021). Seven of eight female participants were taking birth control pills during the data collection period and the timing for their participation was not restricted. The one participant who was not taking birth control pills started the study while in the early follicular phase. All subjects were non-smokers, not obese according to BMI (<30 kg/m^2^), without a history of chronic skin diseases or skin allergies, and not taking medications or supplements that alter cardiovascular or thermoregulatory control.

### Experimental Procedure

**FIGURE 1:**
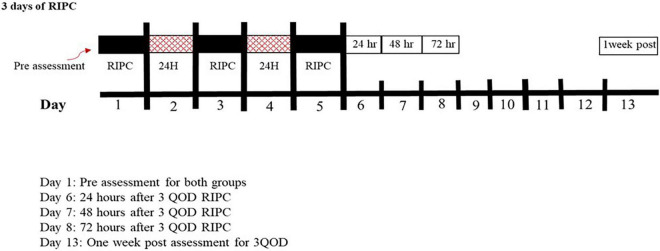
Schematic for the overall experiment procedures for both groups. Black boxes represent RIPC, red boxes represent the break, and white boxes represent microvascular assessments.

This study was designed to assess the temporal component of the skin blood flow responses following 3QOD RIPC. RIPC was elicited by 4 cycles for 5 min of arm cuff inflation to ∼220 mmHg on the dominant arm, separated by 5 min of deflation. Arm blood flow was restricted by a pneumatic cuff (Rapid Cuff Inflation System, D.E. Hokanson, Inc., Bellevue, WA, United States) placed around the upper arm. Figure 1 shows a schematic of this study protocol. The first RIPC session was conducted immediately following the initial assessment of cutaneous microvascular function, and the next two sessions were conducted at 48 ± 2 h intervals. To test our hypothesis, we measured skin blood flow responses to local heating (*T*_loc_ = 42°C) and iontophoresis-mediated drug infusion to the arm contralateral to the ischemic arm. Skin blood flow measurements were repeated in the non-dominant arm five times over 7 days–immediately before the first RIPC bout and then 24 ± 2, 48 ± 2, 72 ± 2, and 168 ± 2 h (i.e., 1 week) after the last bout of RIPC.

On the days of the experiment, subjects were asked to fast (>3 h before coming to the laboratory), abstain from alcohol and caffeine (>12 h), and refrain from either novel or strenuous physical activity (24 h). Upon arrival at the laboratory (*T*_*a*_ = 23°C, RH = 18%), the participants rested on a bed in a semi-reclined position. Three electrodes were placed on the upper body to monitor heart rate continuously *via* a lead II electrocardiograph (CT-1000 cardiotachometer, CWE Inc., Ardmore, PA, United States). Blood pressure was measured every 10–15 min throughout the entire experimental protocol (Critikon 9300 Dinamap XL, Vital Signs Monitor, Tempa, FL, United States.

Skin blood flow of the non-dominant forearm was assessed using laser speckle contrast imaging (LSCI) (Moors FLPI-2, Moor Instruments, Axminster, United Kingdom). The LSCI imager was placed approximately 15–20 cm above the ventral surface of the forearm (wavelength = 785 nm, image exposure time = 4 ms, image acquisition rate = 25 frames/s). A vacuum cushion was placed underneath the measurement forearm to stabilize and minimize movement during image collection. After instrumentation was completed and resting hemodynamic data were collected, local heating and iontophoresis protocols began. The first RIPC session was conducted immediately after the initial assessment of cutaneous microvascular function, and the next two sessions were conducted at 48 ± 2 h intervals.

### Laser Speckle Contrast Imaging

Laser speckle contrast imaging emits a beam of laser light that reflects off red blood cells as they move through skin vessels, thereby providing a non-invasive index of skin blood flow. It provides good to excellent day-to-day reproducibility; coefficient of variation (CV) for the local heating plateau is 15%, Ach peak is 19%, and SNP peak is 15% (Roustit et al., 2010; Tew et al., 2011; Mahe et al., 2012; Loader et al., 2017). Furthermore, we tested a group of participants (*n* = 8) who did not receive RIPC but underwent repeated local heating, Ach, and SNP measurements 2 weeks later, utilizing the same LSCI protocol. CVs were 7, 13, and 12%, respectively (local heating plateau: 1.93 ± 0.21 vs. 1.96 ± 0.17, Ach: 1.44 ± 0.2 vs. 1.44 ± 0.2, SNP: 1.14 ± 0.09 vs. 1.17 ± 0.14 PU⋅mmHg^–1^). Thus, in our laboratory, our LSCI measurements with these perturbations have good to excellent reproducibility, thereby minimizing any effect that might occur due to chance.

### Local Heating

A local heating unit (moor VHS-heat, Moor Instruments, Axminster, United Kingdom) was placed on the forearm skin surface >3 cm away from the antecubital fossa and filled with deionized water. A nickel-sized temperature controller was attached to the non-dominant forearm and maintained at a thermoneutral skin temperature (*T*_loc_ = 33°C or 91°F) during baseline. After collecting baseline measurements for ∼10–15 min, the temperature of the controller was increased at a rate of 0.1°C/s to *T*_loc_ = 42°C and maintained for ∼50 min until a stable plateau in the vasodilation response was achieved. Once the local heating procedure was started, the iontophoresis procedure began.

### Iontophoresis

Iontophoresis was used to non-invasively administer Ach and SNP to test endothelium-dependent and endothelium-independent dilation, respectively. The active and indifferent electrodes were placed 5–10 cm apart on the skin surface of the measurement site. The active electrode was located at a separate skin site to prevent any current-related influence on the vasomotor function of the local heating site. Acetylcholine (Ach) and SNP were mixed with saline solution. To check non-drug-related responses, saline solution was administrated before each drug administration. When the local heating protocol began, saline solution was administrated *via* 20 μA anodal current for 200 s. After saline solution, 2% Ach solution, diluted in saline, was administrated with the same current intensity (20 μA anodal current for 200 s). After hyperemia resolved fully, the active electrode site was relocated to a 2-cm separated skin site and changed the polarity of iontophoresis. Saline solution was also administrated followed by a 1% SNP, diluted in saline, 20 μA cathodal current for 400 s. Those currents and duration were administrated in our previous studies and demonstrated a microvascular response without eliciting current-induced vasodilation (Kim et al., 2020, 2021).

### Data Analysis

Laser speckle contrast imaging data were standardized as CVC, calculated as the mean LSCI flux (PU) divided by MAP (diastolic pressure + 1/3 pulse pressure), and expressed as PU⋅mmHg^–1^. The baseline for all measurements (local heating-, Ach-, and SNP-induced vasodilatation) and the local heating plateau were averaged over a stable 2-min period. The initial peak of local heating and the peak responses to Ach and SNP were the means of 10 s of the highest flux value. Local heating-induced vasodilation, expressed as ΔCVC, was calculated using (local heating plateau CVC–baseline CVC). Both Ach- and SNP-induced vasodilation were also expressed as ΔCVC (Ach peak CVC–baseline CVC; SNP peak CVC–baseline CVC, respectively).

One-way repeated measure ANOVA (pre-RIPC vs. 24 h vs. 48 h vs. 72 h vs. 1 week after 3QOD RIPC as the main effect) was used to assess skin blood flow response changes to local heating, Ach, and SNP (SPSS version 26, IBM, Chicago, IL, United States). Fisher’s LSD *post-hoc* test was used to find significant differences between time points. Statistical significance was set at α = 0.05. Skin blood flow data and subject characteristics are presented as mean ± SD.

## Results

**FIGURE 2:**
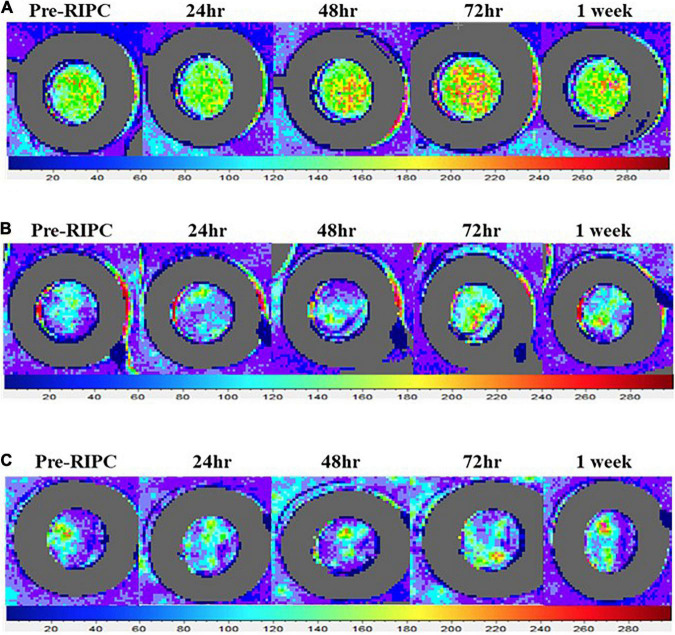
Representative laser speckle contrast images illustrating the increase in the vasodilation responses to local heating **(A)**, acetylcholine **(B)**, and sodium nitroprusside **(C)** following a single bout of RIPC. LSCI measures the extent to which moving red blood cells reflect a beam of the laser. The color palette indicates flux value changes. Increased orange and red in the image indicate greater red blood cell movement detected.

Representative images from local heating (*T*_loc_ = 42°C) and Ach- and SNP-induced cutaneous vasodilation responses throughout each measurement point (pre-RIPC, 24, 48, 72 h, and 1 week) are presented in Figure 2. The orange and red colors in Figure 2 represent skin blood flow augmentation.

Mean arterial pressure and local heating responses are provided in Table 1. It did not change throughout the five measurement visits (*P* = 0.68). Additionally, the CVC at baseline and the initial peak of the heating response did not change between measurement visits (baseline CVC, *P* = 0.13; initial peak CVC, *P* = 0.42). However, the local heating plateau and ΔCVC changed after 3QOD RIPC (main effect, *P* < 0.05). The *post-hoc* test showed both local heating plateau and ΔCVC tended to be increased 24 h after 3QOD RIPC (pre-RIPC vs. 24 h: heating plateau, *P* = 0.054; ΔCVC, *P* = 0.09). After 72 h, RIPC increased at both the local heating-induced plateau and ΔCVC at 72 h (pre-RIPC vs. 72 h: heating plateau, *P* < 0.05; ΔCVC, *P* < 0.05) and tended to remain elevated for 1 week after the last day of 3QOD RIPC (pre-RIPC vs. 1 week: heating plateau, *P* = 0.075; ΔCVC, *P* = 0.054).

**TABLE 1:** Local heating-mediated cutaneous vasodilation following 3 bouts of RIPC performed every other day.

	Pre-RIPC	24 h	48 h	72 h	1 Week
MAP (mmHg)	83.8 ± 6.0	80.5 ± 7.2	81.2 ± 5.2	81.2 ± 6.5	81.5 ± 4.4
Baseline CVC	0.41 ± 0.06	0.45 ± 0.09	0.43 ± 0.08	0.45 ± 0.06	0.41 ± 0.06
Initial Peak	1.41 ± 0.32	1.54 ± 0.39	1.51 ± 0.26	1.55 ± 0.35	1.45 ± 0.31
Heating plateau	1.49 ± 0.26	1.65 ± 0.32^†^	1.60 ± 0.33	1.78 ± 0.48*	1.62 ± 0.39^‡^
Δ CVC	1.08 ± 0.24	1.20 ± 0.28^‡^	1.17 ± 0.31	1.34 ± 0.46*	1.21 ± 0.36^†^

Values are represented as cutaneous vascular conductance (PU⋅mmHg^–1^); *P ≤ 0.05 vs. pre-RIPC, ^†^P = 0.054 vs. pre-RIPC, ^‡^P ≤ 0.09 vs. pre-RIPC.

Cutaneous responses to Ach and SNP iontophoresis are shown in Table 2. The main effect was not significant for either Ach- or SNP-induced ΔCVC throughout the measurement points (main effect, *P* = 0.22), the Ach-induced peak CVC started to increase at 72 h (pre-RIPC vs. 72 h: *P* = 0.06), and both Ach-induced peak CVC and ΔCVC significantly elevated for 1 week after the last day of 3QOD RIPC (pre-RIPC vs. 1 week: *P* ≤ 0.05). The SNP-induced peak and ΔCVC were also increased 24 h after 3QOD of RIPC (pre-RIPV vs. 24 h: *P* < 0.05) but returned to pre-RIPC in subsequent time points (*P* > 0.05).

**TABLE 2:** Acetylcholine- and sodium nitroprusside-mediated cutaneous vasodilation following 3 bouts of RIPC performed every other day.

	Pre-RIPC	24 h	48 h	72 h	1 Week
**Baseline CVC**					
Ach	0.46 ± 0.1	0.50 ± 0.1	0.49 ± 0.1	0.52 ± 0.1	0.52 ± 0.2
SNP	0.55 ± 0.2	0.55 ± 0.1	0.54 ± 0.2	0.52 ± 0.1	0.55 ± 0.1
**Peak**					
Ach	1.20 ± 0.5	1.22 ± 0.5	1.30 ± 0.5	1.40 ± 0.4^†^	1.46 ± 0.6*
SNP	1.01 ± 0.4	1.16 ± 0.4*	1.04 ± 0.4	1.05 ± 0.3	1.15 ± 0.3
**Δ CVC**					
Ach	0.73 ± 0.4	0.85 ± 0.5	0.82 ± 0.4	0.88 ± 0.3	0.95 ± 0.5*
SNP	0.47 ± 0.3	0.63 ± 0.3*	0.52 ± 0.3	0.53 ± 0.2	0.61 ± 0.4

Values are represented as cutaneous vascular conductance (PU⋅mmHg^–1^); *P < 0.05 vs. pre-RIPC, ^†^P = 0.06 vs. pre-RIPC.

**FIGURE 3:**
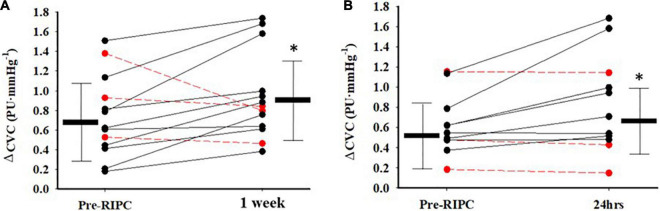
Indival changes in CVC from baseline (ΔPU⋅mmHg^−1^) in response to Acetylcholine **(A)** between Pre-RIPC and a week after 3 days of RIPC, and sodium nitroprusside **(B)** between Pre-RIPC and 24 h after 3 days of RIPC. The red dashed lines represent participants that did not exhibit an increase in dilatory function. Group means ± SD are also shown. *p< 0.05 vs Pre-RIPC.

The individual response in Ach-induced ΔCVC between pre-RIPC and 1 week after 3QOD RIPC is shown in Figure 3A, and the individual response in SNP-induced ΔCVC between pre-RIPC and 24 h after 3QOD RIPC is shown in Figure 3B. For most participants (10 out of 13), Ach- and SNP-induced ΔCVC increased after 1 week and 24 h after 3QOD RIPC, respectively.

## Discussion

The purpose of this study was to test the extent to which 3QOD RIPC improves both endothelium-dependent and endothelium-independent cutaneous vasodilation and whether these changes persisted a week thereafter. The major findings were as follows: (1) 3QOD RIPC improved local heating-induced cutaneous vasodilation 72 h later, with the effect lasting 1 week; (2) Ach-induced cutaneous vasodilation increased and was significantly higher 1 week after 3QOD; and (3) SNP-mediated vasodilation was transiently increased 24 h before returning to baseline. These data suggest that 3QOD RIPC induced a delayed improvement in endothelium-dependent cutaneous vasodilation that persisted for at least 1 week. However, 3QOD was not sufficient to elicit lasting effects in the endothelium-independent vasodilation response.

A single bout of RIPC exhibits a characteristic delayed protective response that lasts for ∼24–72 h (Bolli et al., 2007). We have recently established that this delayed response also occurs in the human cutaneous microvasculature (Kim et al., 2021). During this phase, there is an increase in the synthesis of heat stress proteins (HSP 60 and HSP 72), superoxide dismutase (SOD), and inducible nitric oxide synthase (Marber et al., 1993; Yamashita et al., 1994; Wang et al., 2002; Doeppner et al., 2018), which may augment endothelial cell function (Bolli et al., 2007; Thijssen et al., 2016). In this study, the RIPC intervention was designed to stimulate the delayed phase by timing the implementation of subsequent RIPC sessions with the peak response (∼48 h) seen after the previous RIPC bout (Kim et al., 2021). Thus, perhaps the third RIPC bout elicits a greater or more prolonged delayed window effect than what is typically observed after the first RIPC bout. Our findings support this notion in that 3QOD RIPC improved endothelium-dependent cutaneous vasodilation that persisted for a week.

Both local heating- and Ach-induced cutaneous vasodilation elicit endothelium-mediated dilation *via* nitric oxide (NO), endothelium-derived hyperpolarization factors (EDHF), and prostanoids (Noon et al., 1998; Kellogg et al., 1999, 2005; Durand et al., 2004; Minson, 2010; Brunt and Minson, 2012; Choi et al., 2014; Brunt et al., 2015). Both perturbations increase NO, which contributes to ∼50% of the total cutaneous vasodilation response (Fujii et al., 2013; Choi et al., 2014; Brunt et al., 2015; Greaney et al., 2019). Since NO is quickly inactivated with oxidative stress (Lobo et al., 2010), the elevated SOD level (Monk et al., 1989) during the delayed phase following RIPC may protect eNOS and elevate NO bioavailability. Furthermore, elevated NO bioavailability directly induces endothelium-dependent cutaneous vasodilation and stimulates K_ATP_ channels that induced EDHF-mediated vasodilation (Fujii et al., 2020). However, our previous study has shown that 7 consecutive days of RIPC did not improve NO contribution on local heating cutaneous vasodilation even after 1 week of the last bout of RIPC (Lang et al., 2019). Therefore, NO bioavailability elevations in the delayed window may contribute to prolonged endothelium-dependent cutaneous vasodilation improvements by stimulating EDHF *via* K_ATP_ channels (Fujii et al., 2020). This is supported by animals studies showing that the activity of biosynthetic enzymes for hydrogen sulfide increases during the delayed window (Wang et al., 2021).

Although both local heating and Ach-mediated dilation were increased following 3QOD RIPC, the increase in the timing of their CVC differed. Ach elicited more gradual improvements in the dilation response through the measurement periods that reached significance 1-week post-RIPC. This may be explained by our method of Ach administration and the lower drug concentration that was used. Higher doses of Ach (≥10 mM) administered by intradermal microdialysis showed a greater EDHF contribution to the vasodilation response (Brunt et al., 2015). Therefore, it is possible that our Ach concentration may not be robust enough to stimulate EDHF throughout the measurement points. In contrast, EDHF strongly contributes to local heating-mediated vasodilation (Brunt and Minson, 2012; Choi et al., 2014; Brunt et al., 2015), which showed an earlier and more robust increase following 3QOD RIPC. However, further study is required to confirm the role of EDHF on the effects of repeated RIPC.

Endothelium-independent cutaneous vasodilation may be explained by vascular smooth muscle sensitivity to NO or angiogenesis. NO activates myosin light chain phosphatase *via* cyclic guanosine monophosphate (cGMP) and induces smooth muscle relaxation. A previous study has shown circulating cGMP level augmentation after ischemic preconditioning on an animal *in vivo* heart (Iliodromitis et al., 1996). In humans, vascular endothelial growth factors (VEGF) and endothelial progenitor cells contribute to new blood vessel formation following 4 weeks of repeated RIPC (Kimura et al., 2007). Furthermore, we showed that 2 weeks of repeated RIPC improved endothelium-independent cutaneous vasodilation (Kim et al., 2020). Therefore, we hypothesized that 3QOD RIPC may also augment the endothelium-independent vasodilation response; however, we observed an increase in the endothelium-independent vasodilation 24 h after 3QOD RIPC that did not persist for a week. These results suggested that 3QOD RIPC may transiently improve vascular smooth muscle sensitivity but may not be sufficient enough to elicit cutaneous microvascular angiogenesis.

Multiple repeated RIPC studies have used different intervention durations, ranging from 1 to 8 weeks (Kimura et al., 2007; Jones et al., 2014, 2015; Lang et al., 2019; Kim et al., 2020). One of the collective findings from these studies was that longer durations of RIPC intervention did not elicit greater local heating-mediated endothelium-dependent cutaneous vasodilation (Jones et al., 2015; Kim et al., 2020). We extended these findings to demonstrate that 3QOD RIPC bouts were sufficient to elicit lasting improvement in the local heating-mediated dilation response. Furthermore, since a single bout of RIPC elicits a characteristic delayed response (Kim et al., 2021), the pattern of when RIPC is administered may also influence cutaneous microvascular function. From a clinical perspective, it is important to know the intervention duration and frequency needed to elicit sustained responses to optimize the beneficial or protective effects of RIPC without unnecessary pain to the clinical population.

### Limitations

Although 3QOD RIPC statistically improved endothelium-mediated cutaneous vasodilation, a few of the participants did not respond in this manner (i.e., 3 out of 13 subjects–1 male, 2 females, Figure 3). Since 3QOD RIPC bouts spanned only a week, the individual response pattern may vary due to individual characteristics such as sex, physical fitness level, and blood lipid level (Trachte et al., 2020). Our sample size was not large enough to detect differences based on these characteristics. Regarding sex, there were more women (*n* = 8) than men (*n* = 5) in the study, which may affect the generalizability of the results in this study. However, neither this study nor the previous studies examining the effects of RIPC in skin circulation have suggested that sex differences in vascular reactivity occur (Deschênes et al., 2016; Lang et al., 2019; Kim et al., 2020, 2021). Thus, data in men and women were pooled in this current study for analysis. Finally, this study did not have a time control group. However, we previously measured interday reproducibility of skin blood flow responses to local heating, Ach, and SNP with LSCI. Our skin blood flow measurement with LSCI showed good reproducibility (C.V. < 13%) compared with previous studies (C.V. < 30%) (Tew et al., 2011; Mahe et al., 2012). Thus, our current data are reliable to confirm our results.

## Conclusion

Compared with a single bout of RIPC, as shown in our previous study (Kim et al., 2021), 3QOD elicits more sustained improvement in microvascular function. 3QOD RIPC induced delayed endothelium-dependent cutaneous vasodilation, and this improvement remained elevated for 1 week after the last RIPC bout. However, endothelium-independent vasodilation was transiently elevated for 24 h after RIPC and did not persist. Our findings indicate that repeated RIPC administered every other day, which corresponds to the peak of the delayed phase, induces more sustained endothelium-mediated function improvements. This suggests that an every other day RIPC protocol may optimize endothelial function, and more RIPC bouts are needed to elicit cutaneous microvascular structural changes.

## Data Availability Statement

The raw data supporting the conclusions of this article will be made available by the authors, without undue reservation.

## Ethics Statement

The studies involving human participants were reviewed and approved by the Iowa State University Institutional Review Board. The patients/participants provided their written informed consent to participate in this study.

## Author Contributions

JK, WF, and JL contributed to the conception and design of the study, interpreted the data, and edited and revised the manuscript. JK collected and analyzed data, and drafted the manuscript. All authors contributed to the article and approved the submitted version.

## Conflict of Interest

The authors declare that the research was conducted in the absence of any commercial or financial relationships that could be construed as a potential conflict of interest.

## Publisher’s Note

All claims expressed in this article are solely those of the authors and do not necessarily represent those of their affiliated organizations, or those of the publisher, the editors and the reviewers. Any product that may be evaluated in this article, or claim that may be made by its manufacturer, is not guaranteed or endorsed by the publisher.

## Abbreviations

RIPC: remote ischemic preconditioning
CV: coefficient of variation
BMI: body mass index
CVC: cutaneous vascular conductance
MAP: mean arterial pressure
Ach: acetylcholine
SNP: sodium nitroprusside
VEGF: vascular endothelial growth factor
IR: ischemia-reperfusion
EDHF: endothelium-derived hyperpolarization factor
NO: nitric oxide
SOD: superoxide dismutase
3QOD: 3 bouts of RIPC performed every other day.
